# Brain-Derived Neurotrophic Factor (BDNF) Role in Cannabinoid-Mediated Neurogenesis

**DOI:** 10.3389/fncel.2018.00441

**Published:** 2018-11-28

**Authors:** Filipa Fiel Ferreira, Filipa F. Ribeiro, Rui S. Rodrigues, Ana Maria Sebastião, Sara Xapelli

**Affiliations:** ^1^Instituto de Farmacologia e Neurociências, Faculdade de Medicina, Universidade de Lisboa, Lisbon, Portugal; ^2^Instituto de Medicina Molecular João Lobo Antunes, Faculdade de Medicina, Universidade de Lisboa, Lisbon, Portugal

**Keywords:** postnatal neurogenesis, subventricular zone, dentate gyrus, cannabinoid receptors, brain-derived neurotrophic factor

## Abstract

The adult mammalian brain can produce new neurons in a process called adult neurogenesis, which occurs mainly in the subventricular zone (SVZ) and in the hippocampal dentate gyrus (DG). Brain-derived neurotrophic factor (BDNF) signaling and cannabinoid type 1 and 2 receptors (CB1R and CB2R) have been shown to independently modulate neurogenesis, but how they may interact is unknown. We now used SVZ and DG neurosphere cultures from early (P1-3) postnatal rats to study the CB1R and CB2R crosstalk with BDNF in modulating neurogenesis. BDNF promoted an increase in SVZ and DG stemness and cell proliferation, an effect blocked by a CB2R selective antagonist. CB2R selective activation promoted an increase in DG multipotency, which was inhibited by the presence of a BDNF scavenger. CB1R activation induced an increase in SVZ and DG cell proliferation, being both effects dependent on BDNF. Furthermore, SVZ and DG neuronal differentiation was facilitated by CB1R and/or CB2R activation and this effect was blocked by sequestering endogenous BDNF. Conversely, BDNF promoted neuronal differentiation, an effect abrogated in SVZ cells by CB1R or CB2R blockade while in DG cells was inhibited by CB2R blockade. We conclude that endogenous BDNF is crucial for the cannabinoid-mediated effects on SVZ and DG neurogenesis. On the other hand, cannabinoid receptor signaling is also determinant for BDNF actions upon neurogenesis. These findings provide support for an interaction between BDNF and endocannabinoid signaling to control neurogenesis at distinct levels, further contributing to highlight novel mechanisms in the emerging field of brain repair.

## Introduction

Constitutive neurogenesis occurs in the adult mammalian brain where NSPC are able to differentiate into three neural lineages, neurons, astrocytes and oligodendrocytes (Gage, 2000; Gross, 2000). These multipotent cells exhibit properties of self-renewal and cell proliferation that allow the maintenance of their own pool (Ma et al., 2009). Neurogenesis occurs mainly in two brain areas, the subventricular zone (SVZ) and the subgranular zone (SGZ) within the DG of the hippocampus. These regions are packed with NSPC that originate neuroblasts which migrate toward their final destinations, where they differentiate into mature neurons and are integrated into the neuronal circuitry (Lledo et al., 2006; Zhao et al., 2008; Ming and Song, 2011).

Adult neurogenesis and the neurogenic niches are highly regulated by several factors (intrinsic and extrinsic factors) that control the NSPC rates of proliferation, lineage differentiation, migration, maturation and survival (Ming and Song, 2011). Knowing and understanding the actions of these factors will further contribute to develop new therapeutic strategies useful for brain repair and regeneration. However, there is still a lack of knowledge regarding the key factors that regulate each step of postnatal neurogenesis.

The role of neurotrophins and, in particular, brain-derived neurotrophic factor (BDNF) in adult neurogenesis has been the subject of many studies (Henry et al., 2007; Chan et al., 2008; Vilar and Mira, 2016). BDNF is expressed in both SVZ and SGZ neurogenic niches (Galvão et al., 2008; Li et al., 2008) but its precise role in adult neurogenesis is still not consensual. In fact, some studies suggest that BDNF is important to positively regulate DG cell proliferation and survival (Chan et al., 2008; Li et al., 2008) while others report no BDNF-induced changes in DG neurogenesis (Choi et al., 2009). In SVZ, most studies depict that BDNF does not promote any significant changes in cell proliferation and survival (Henry et al., 2007; Galvão et al., 2008), despite having a role in the migration of SVZ-derived cells (Snapyan et al., 2009; Bagley and Belluscio, 2010). Despite the available contradictory data, BDNF, through TrkB signaling, was shown to have an essential role in the regulation of dendritic complexity as well as synaptic formation, maturation and plasticity of newborn neurons (Chan et al., 2008; Gao et al., 2009; Wang et al., 2015).

Besides expressing BDNF, NSPC present in the neurogenic niches were shown to express all the elements of the endocannabinoid system (Aguado et al., 2005; Arévalo-Martín et al., 2007), including the main cannabinoid receptors type 1 (CB1R) and type 2 (CB2R) receptors (Rodrigues et al., 2017). They are both present in the CNS, although CB2R expression is relatively higher in the immune system (Galve-Roperh et al., 2007). In recent years, the role of cannabinoids in neurogenesis has been of particular interest given their multiplicity of neuromodulatory functions (Mechoulam and Parker, 2013). Cannabinoid receptors modulate adult neurogenesis by acting at distinct neurogenic phases (Prenderville et al., 2015). Importantly, activation of type 1 (Xapelli et al., 2013) or type 2 cannabinoid receptors (Palazuelos et al., 2006) by selective agonists was found to regulate cell proliferation, neuronal differentiation and maturation (Rodrigues et al., 2017).

Several studies have provided molecular and functional evidence for a crosstalk between BDNF and endocannabinoid signaling (Maison et al., 2009; Zhao et al., 2015). Synergism between BDNF and CB1R has been observed both *in vitro* and *in vivo* (De Chiara et al., 2010; Galve-Roperh et al., 2013). In particular, BDNF was shown to regulate striatal CB1R actions (De Chiara et al., 2010). Moreover, evidence for BDNF-TrkB signaling interplay with CB1R has been shown to trigger endocannabinoid release at cortical excitatory synapses (Yeh et al., 2017). Importantly, genetic deletion of CB1R was shown to promote a decrease in BDNF expression (Aso et al., 2008) while induction of BDNF expression contributed to the protective effect of CB1R activity against excitotoxicity (Marsicano, 2003; Khaspekov et al., 2004). Moreover, CB1R activity can enhance TrkB signaling partly by activating MAP kinase/ERK kinase pathways (Derkinderen et al., 2003) but also by directly transactivating the TrkB receptors (Berghuis et al., 2005). Δ^9^-THC, the principal active component of cannabis, was shown to promote upregulation of BDNF expression (Butovsky et al., 2005) whereas increased levels of BDNF were shown to rescue the cognitive deficits promoted by Δ^9^-THC administration (Segal-Gavish et al., 2017). Interestingly, clinical data suggests that acute and chronic intermittent exposure to Δ^9^-THC alters BDNF serum levels in humans (D’Souza et al., 2009).

Given the evidence that BDNF and cannabinoid signaling can affect neurogenesis as well as the fact that BDNF may interact with cannabinoid receptors, we hypothesized that cannabinoid receptors could act together with BDNF signaling to fine-tune neurogenesis. We show for the first time that endogenous BDNF is crucial for the cannabinoid-mediated effects on SVZ and DG neurogenesis to happen. Moreover, we demonstrate that CB2R has a preponderant role in regulating some of the BDNF actions on neurogenesis. Taken together, our results suggest an important crosstalk between BDNF and cannabinoid signaling to modulate postnatal neurogenesis.

## Materials and Methods

### Ethics

All experiments were performed in accordance with the European Community (86/609/EEC; 2010/63/EU; 2012/707/EU) and Portuguese (DL 113/2013) legislation for the protection of animals used for scientific purposes. The protocol was approved by the “iMM’s institutional Animal Welfare Body – ORBEA-iMM and the National competent authority – DGAV (Direcção Geral de Alimentação e Veterinária).” The work was performed with biological material obtained from rat pups and subsequently maintained *in vitro*. The pups were handled according to standard and humanitarian procedures to reduce animal suffering.

### SVZ and DG Cell Cultures

SVZ and DG neurospheres were prepared from early postnatal (P1-3) Sprague-Dawley rats. SVZ and DG fragments were dissected out from 450 μm-thick coronal brain slices, digested with 0.05% Trypsin-EDTA (Life Technologies, Carlsbad, CA, United States) in Hank’s balanced saline solution (HBSS, Life Technologies), and mechanically dissociated with a P1000 pipette. The originated cell suspension was then diluted in serum-free medium (SFM), composed of Dulbecco’s modified Eagle’s medium/Ham’s F-12 medium with glutaMAX (DMEM+GlutaMAX, Life Technologies) supplemented with 100 U/mL penicillin and 100 μg/mL streptomycin (Pen/Strep; Life Technologies), 1% B27 (Life Technologies) and growth factors (for SVZ cells: 20 ng/mL epidermal growth factor (EGF; Life Technologies); for DG cells: 20 ng/mL epidermal growth factor (EGF; Life Technologies) and 10 ng/mL fibroblast growth factor-2 (FGF-2; Life Technologies) (proliferative conditions). SVZ cells were then plated on uncoated Petri dishes and allowed to develop for 6 days, whereas DG cells were allowed to develop for 10 days, both in a 95% air-5% CO2 humified atmosphere at 37°C. Six-day-old SVZ neurospheres and 10-day-old DG neurospheres were adhered for 24 h onto glass coverslips coated with 0.1 mg/mL poly-D-lysine (PDL, Sigma-Aldrich, St. Louis, MO, United States) in SFM devoid of growth factors (differentiative conditions). Two days after plating, the medium was renewed with or without (control) a range of pharmacological treatments (see Table 1).

**Table 1:** Pharmacological treatments used.

Drug	Chemical name	Concentration used	Catalog number	*K_i_* value, nM (according to Pertwee, 2008)	Company
**WIN55,212-2** [(R)-(+)-[2,3-Dihydro-5-methyl-3-(4-morpholinylmethyl)pyrrolo[1,2,3-de]-1,4-benzoxazin-6-yl]-1-naphthalenylmethanone]	Cannabinoid receptor CB_1_ or CB_2_ non-selective agonist	1 μM	1038	1.89–123 for CB1R or 0.28–16.2 for CB2R	Tocris, Bristol (United Kingdom)
**ACEA** [*N*-(2-Chloroethyl)-5*Z*,8*Z*,11*Z*,14*Z*-eicosatetraenamide]	Cannabinoid CB_1_ receptor selective agonist	1 μM	1319	1.4 for CB1R	
**HU-308** [4-[4-(1,1-Dimethylheptyl)-2,6-dimethoxyphenyl]-6,6-dimethylbicyclo[3.1.1]hept-2-ene-2-methanol]	Cannabinoid CB_2_ receptor selective agonist	1 μM	3088	22.7 for CB2R	
**AM251** [*N*-(Piperidin-1-yl)-5-(4-iodophenyl)-1-(2,4-dichlorophenyl)-4-methyl-1*H-*pyrazole-3-carboxamide]	Cannabinoid CB_1_ receptor selective antagonist	1 μM	1117	7.49 for CB1R	
**AM630** [6-Iodo-2-methyl-1-[2-(4-morpholinyl)ethyl]-1*H*-indol-3-yl](4-methoxyphenyl)methanone]	Cannabinoid CB_2_ receptor selective antagonist	1 μM	1120	31.2 for CB2R	
**BDNF**	TrkB ligand	30 ng/mL	–	0.99 for TrkB (according to Hempstead et al., 1991)	Kind gift from Regeneron Pharmaceuticals (Tarrytown, NY, United States)
**TrkB-Fc**	BDNF scavenger	2 μg/mL	688-TK	NA	R&D Systems (Minneapolis, MN, United States)

### Pharmacological Treatments

To investigate the crosstalk between CB1R, CB2R and BDNF on cell-fate, cell proliferation and neuronal differentiation CB1R selective agonist (ACEA, 1 μM), CB2R selective agonist (HU-308, 1 μM), non-selective cannabinoid receptor agonist WIN55,212-2 (1 μM) or BDNF (30 ng/mL) were incubated in SVZ and DG cell cultures. Moreover, selective antagonists for CB1R (AM251, 1 μM) and CB2Rs (AM630, 1 μM) or scavenger for BDNF (TrkB-Fc, 2 μg/mL) were used (Table 1). TrkB-Fc chimera consists of an extracellular domain of human TrkB fused to the C-terminal Fc region of human IgG1 used to bind to BDNF, therefore, removing available BDNF in the media. The ligand concentrations used in the studies were selected from previous published work (Rodrigues et al., 2017).

To study cell-fate, a Sox2 cell-pair assay was performed as described by Xapelli et al. (2013), where dissociated SVZ and DG cell suspensions obtained during the cell culture procedure were plated on poly-D-lysine coated glass coverslips at a density of 12800 cells/cm^2^ and 19200 cells/cm^2^, respectively. After seeding, SVZ and DG cells were grown, respectively, in SFM supplemented with 10 ng/mL EGF (low EGF) and in SFM supplemented with 10 ng/mL EGF and 5 ng/mL FGF-2 (low EGF/FGF-2). Moreover, plated cells were treated for 24 h with the drugs that modulate CB1R and CB2R and, BDNF (Table 1).

To study cell proliferation, plated neurospheres in differentiative conditions were allowed to develop for 48h in the absence (control) or presence with the aforementioned drugs (Table 1).

Neuronal differentiation was assessed by allowing neurospheres to develop for 7 days in the absence (control) or presence of the drugs (Table 1).

Whenever cultures needed to be co-treated with a combination of drugs, treatment with selective antagonists for CB1Rs and CB2Rs or TrkB-Fc was performed 30 min prior to the treatment with the CB1R or CB2R selective agonists or BDNF.

### Cell Commitment Study (Cell-Pair Assay)

Dissociated SVZ or DG cells that were treated for 24 h with the drugs were fixed in phosphate-buffered saline (PBS) containing 4% paraformaldehyde (PFA) for 30 min and the stained for Sox2 (Table 2), a marker of NSPC with the ability to self-renewal. Cell pairs resulting from the division of a single NSPC were counted and categorized in 3 groups according to their Sox 2 expression: in both daughter cells (Sox2 +/+ cell pairs), in only one of the daughter cell (Sox2 +/- cell pairs) and no expression (Sox2 -/- cell pairs). Sox2 expression in the daughter cells characterizes the response of cells to the pharmacological treatment applied, ultimately reflecting the cell-fate of the pool of NSPC, namely expansion (symmetrical self-renewal), maintenance (asymmetrical self-renewal) or extinction (symmetrical commitment) (Xapelli et al., 2013).

**Table 2:** Antibodies used for immunocytochemistry.

Antigen	Company	Catalog number	Host	Dilution
**Primary antibodies**				
Sox2 (a marker of neural stem cells with the ability to self-renew)	Santa Cruz Biotechnology (Dallas, TX, United States)	sc-17320	Goat	1:100
BrdU (5-bromo-2′-deoxyuridine)	AbD Serotec, Bio-Rad Laboratories (Oxford, United Kingdom)	OBT00306	Rat	1:200
Neuronal Nuclei (NeuN) (mature neuronal marker)	Cell Signaling Technology (Danvers, MA, United States)	12943	Rabbit	1:200
**Secondary antibodies**				
Anti-Goat Alexa Fluor^®^ 568	Thermo Fisher Scientific (Rockford, IL, United States)	A-11057	Donkey	1:200
Anti-Rat Alexa Fluor^®^ 488	Thermo Fisher Scientific (Rockford, IL, United States)	A-21208	Donkey	1:200
Anti-Rabbit Alexa Fluor^®^ 568	Thermo Fisher Scientific (Rockford, IL, United States)	A-10042	Donkey	1:200

### Cell Proliferation Study

To investigate the effect of the different pharmacological treatments on cell proliferation, SVZ and DG cells were exposed to 10 μM 5-bromo-2^′^-deoxyuridine (BrdU) (Sigma-Aldrich), a synthetic thymidine analog able to substitute thymidine in the DNA double chain synthesis occurring in dividing cells, for the last 4 h of each specific pharmacological treatment (48 h). Then, SVZ and DG cells were fixed in 4% PFA for 30 min and rinsed with PBS at room temperature (RT). Subsequently, BrdU was unmasked by permeabilizing cells in PBS 1% Triton X-100 at RT for 30 min and DNA was denaturated in 1 M HCl for 40 min at 37°C. Following incubation in PBS with 0.5% Triton X-100 and 3% bovine serum albumin (BSA) to block non-specific binding sites, cells were incubated overnight with the anti-rat BrdU antibody (Table 2). After an additional rinse in PBS, nuclei counterstaining and mounting were performed as described previously.

### Cell Differentiation Study

SVZ and DG neurosphere-derived cells treated for 7 days with the drugs were fixed for 30 min in 4% PFA in PBS, permeabilized and blocked for non-specific binding sites for 1h30 with 0.5% Triton X-100 (Sigma-Aldrich) and 6% BSA in PBS. Cells were then incubated overnight at 4°C with the antibody anti-neuronal nuclei (NeuN), a marker of mature neurons (Table 2) in 0.1% Triton X-100 and BSA 0.3% (w/v) in PBS, and then for 1 h at RT with the appropriate secondary antibody (Table 2) in PBS. Nuclei were stained with Hoechst 33342 (6 μg/mL in PBS, Life Technologies). The final preparations were mounted using Mowiol fluorescent medium.

### Microscopy

Fluorescence images were captured using an AxioCamMR3 monochrome digital camera (Carl Zeiss Inc., Göttingen, Germany) mounted on an Axiovert 200 inverted widefield fluorescence microscope (Carl Zeiss Inc.), with a 40x objective. Images were recorded using the software AxioVision 4 (Carl Zeiss Inc.). The pixel size in the object space was 0.25 μm and the captured image size was 1388 × 1040 pixels. Images were stored and analyzed in an uncompressed 8-bit Tiff format.

### Statistical Analysis

In all experiments, measurements were performed at the border of SVZ and DG neurospheres, where migrating cells form a pseudo-monolayer of cells. In every independent experiment, each condition was measured in triplicate, i.e., in three different coverslips. Percentages of Sox2 cell pairs were obtained from counting 60 cell pairs for each condition obtained from 5-9 independent cultures. Percentages of BrdU and NeuN immunoreactive cells were calculated from cell counts in five independent microscopic fields per coverslip with a 40x objective (approximately 200–400 cells per field).

All experiments were analyzed in a double-blind fashion and obtained data was normalized to each corresponding control. Data are expressed as mean ± standard error of the mean (SEM). Statistical significance was determined using one-way analysis of variance followed by Bonferroni’s-multiple comparison test, with *P* < 0.05 considered to represent statistical significance.

## Results

Neurospheres were used as a model to study postnatal neurogenesis dynamics. They consist of spheroid clones of NSPCs that express both Sox2 and Nestin (markers expressed by self-renewing neural precursor cells) and that are able to differentiate into neurons, expressing immature neuronal markers, such as doublecortin and βIII tubulin and mature neuronal markers, such as NeuN (Rodrigues et al., 2017). Furthermore, during neuronal differentiation, these cells start to express phenotypic markers such as vesicular GABA transporter (VGAT, marker for GABAergic neurons) and tyrosine hydroxylase (TH, marker for dopaminergic neurons) in the case of SVZ-derived neurons and VGAT and Vesicular Glutamate transporter 1 (VGlut1, marker for glutamatergic neurons) in the case of DG-derived neurons (Rodrigues et al., 2017). Importantly, SVZ and DG neurospheres were shown to express both CB1R and CB2R throughout the process of differentiation at DIV 1 and DIV 7 as well as in adult tissue (Rodrigues et al., 2017).

### BDNF-CB2R Interaction Regulates Self-Renewal in SVZ Cell Cultures

**FIGURE 1:**
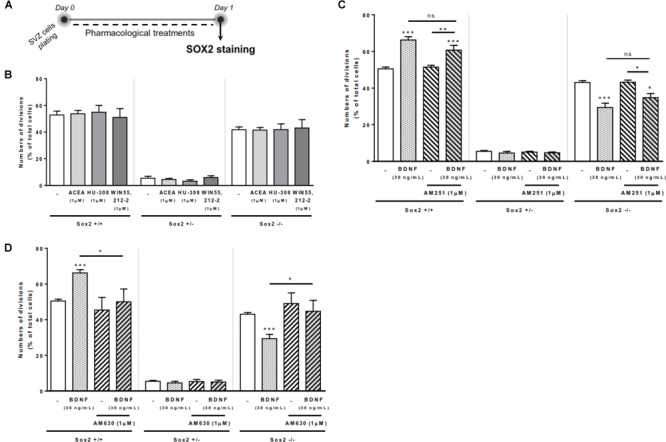
BDNF-CB2R interaction regulates SVZ self-renewal. BDNF treatment promoted an increase in self-renewing capacity of SVZ cells which was blocked by CB2R antagonism, albeit cannabinoid receptor activation had no effect. **(A)** Schematic representation of the experimental protocol used to study cell-fate. Day 0 represents the day of cultures where the SVZ cell suspension was treated with the drugs for 24 h. **(B–D)** Bar graphs depict the percentage of Sox2+/+, Sox2+/–, Sox2–/– cell pairs expressed as percentage of total cells per culture. Data are expressed as mean ± SEM. *n* = 3–9. *^∗^p* < 0.05, *^∗∗^p* < 0.01 and *^∗∗∗^p* < 0.001 using Dunnett’s multiple comparison test. ns, non-significant.

To investigate the ability of BDNF and cannabinoid receptor ligands to modulate the cell-fate of SVZ cells, a Sox2 cell-pair assay was performed in SVZ cells plated for 24 h in medium supplemented or not (control) with receptor ligands (Figure 1A). Cell pairs resulting from the division of a single NSPC were counted and categorized in 3 groups according to their Sox 2 expression: Sox2+/+ cell pairs indicative of pool expansion through symmetrical self-renewal, Sox2+/- cell pairs, indicative of pool maintenance through asymmetrical self-renewal and Sox2-/- cell pairs, indicative of pool extinction through symmetrical commitment. In SVZ cells, neither selective agonists for CB1R (ACEA, 1 μM) or CB2R (HU-308, 1 μM), nor the non-selective cannabinoid receptor agonist, WIN 55,212-2 (1 μM), modified the percentages of either Sox2+/+ cell pairs (Figure 1B).

SVZ cells treated with BDNF (30 ng/mL) showed a significant increase in the percentages of Sox2+/+ cell pairs (66.2 ± 1.78% [95% CI: 62.1–70.3%]; *n* = 9, *p* < 0.001 vs. control) with a concomitant decrease in the percentage of Sox2-/- cell pairs (29.4 ± 2.33% [95% CI: 24.1–34.8%]; *n* = 9, *p* < 0.001 vs. control) (Figures 1C,D), indicating that BDNF is promoting self-renewal of SVZ cells. We next evaluated whether the action of BDNF depends on cannabinoid receptors. SVZ cells were treated with either the CB1R antagonist, AM251 (1 μM), or the CB2R antagonist, AM630 (1 μM), 30 min prior to BDNF treatment and then grown for 24 h in the presence of BDNF (30 ng/mL). The presence of the CB1R antagonist did not block the BDNF-induced effect on SVZ cell-fate (Figure 1C). Remarkably, the increase in the percentage of Sox2+/+ SVZ cell pairs promoted by BDNF treatment was blocked by the presence of the CB2R selective antagonist, AM630 (1 μM) (50.1 ± 7.18% [95% CI: 30.1–70.0%]; *n* = 5, *p* < 0.05 vs. BDNF alone). Similarly, CB2R blockage abolished BDNF-mediated decrease in the percentage of Sox2-/- cell pairs (44.7 ± 6.14%, [95% CI: 27.6–61.7%]; *n* = 5, *p* < 0.05 vs. BDNF alone) (Figure 1D), showing a preponderant role of CB2R in modulating the BDNF actions upon SVZ cell fate. Treatment with selective receptor antagonists alone did not alter SVZ cell-fate (Figures 1C,D).

Altogether, the above data indicate that CB2R modulation interferes with BDNF signaling in regulating SVZ cell-fate.

### CB1R-Induced SVZ Cell Proliferation Is Dependent on Endogenous BDNF

**FIGURE 2:**
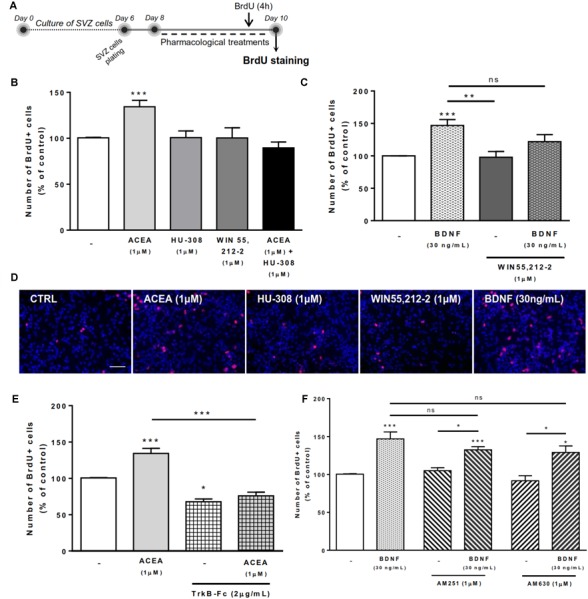
CB1R-induced SVZ proliferation is dependent on endogenous BDNF. SVZ proliferation was increased by CB1R activation and treatment with BDNF. BDNF was required for CB1R-mediated effect to occur. Conversely, BDNF-mediated effect was independent of CB1R or CB2R modulation. **(A)** Schematic representation of the experimental protocol. Day 0 represents the day of cultures; at Day 6 SVZ neurospheres were plated for 48 h and at Day 8 cells were exposed to pharmacological treatments for further 48 h (Day 10). **(B,C,E,F)** Bar graphs depict the number of BrdU-positive cells. Values were normalized to the control mean for each experiment and are represented as mean ± SEM. Control was set to 100%. *n* = 3-19. ^∗^*p* < 0.05 and ^∗∗∗^*p* < 0.001 using Dunnett’s multiple comparison test. ns, non-significant. **(D)** Representative fluorescent digital images of cells immunopositive for BrdU (in red) and Hoechst 33342 staining (blue nuclei). Scale bar = 50 μm.

Next it was investigated whether CB1R/CB2R activation and BDNF could modulate SVZ cell proliferation. For that SVZ cells were treated with selective ligands for 48 h and BrdU was added during the last 4h of the culture to label SVZ cells that went through S-phase. After fixation, incorporated BrdU was immunolabeled and the percentage of positive nuclei was determined (Figure 2A).

As previously described by our group (Rodrigues et al., 2017), treatment of SVZ cells with CB1R agonist ACEA (1 μM) promoted a substantial increase in the number of BrdU-positive cells when compared to control cultures (control: 100.5 ± 0.53% [95% CI: 99.4–101.7%]; ACEA 1 μM: 134.3 ± 6.96% [95% CI: 119.5–149.2%]; *n* = 16, *p* < 0.001) whereas treatment with CB2R agonist HU-308 (1 μM) and cannabinoid non-selective receptor agonist WIN 55,212-2 (1 μM) induced no significant alterations in the number of BrdU-positive cells when compared to control cultures (Figures 2B,D).

We next sought to investigate the combined actions of BDNF and cannabinoid receptor activation on SVZ cell proliferation. We observed that incubation with exogenous BDNF promoted a significant increase in the number of SVZ BrdU-positive cells (BDNF 30 ng/mL: 146.9 ± 9.17% [95% CI: 127.6–166.2%]; *n* = 19, *p* < 0.001) and that this increase was maintained when co-incubating with cannabinoid non-selective receptor agonist WIN 55,212-2 (BDNF 30 ng/mL+WIN 55,212-2 1 μM: 121.9 ± 10.9% [95% CI: 74.8–168.8%]; *n* = 3), although incubation with WIN 55,212-2 *per se* did not affect SVZ cell proliferation (Figures 2C,D).

To evaluate the influence of endogenous BDNF on cannabinoid-mediated SVZ cell proliferation, we used a BDNF scavenger (TrkB-Fc chimera, 2 μg/mL). The incubation with the scavenger alone caused a significant decrease in the percentage of BrdU-positive cells (TrkB-Fc 2 μg/mL: 67.6 ± 3.85% [95% CI: 51.1–84.3%]; *n* = 3, *p* < 0.05 vs. control) (Figure 2E), indicating a preponderant role of endogenous BDNF upon cell proliferation. The presence of the scavenger abolished the enhancement in BrdU-positive cells caused by CB1R agonist, ACEA (1 μM) (Figure 2E), indicating not only that SVZ cell proliferation is modulated by CB1R but that this modulation is dependent on endogenous BDNF.

Interestingly, in the presence of the selective CB1R or CB2R antagonists (AM251 and AM630, respectively) the increase in cell proliferation mediated by BDNF was not changed (*p* > 0.05 vs. BDNF, Figure 2F). These data suggest that BDNF plays a crucial role in regulating SVZ cell proliferation and that endogenous BDNF availability is required for CB1R actions upon this process. However, the effect mediated by BDNF in SVZ cell proliferation is not dependent on CB1R or CB2R.

### BDNF Crosstalk With Cannabinoid Receptors Modulates Neuronal Differentiation at SVZ

**FIGURE 3:**
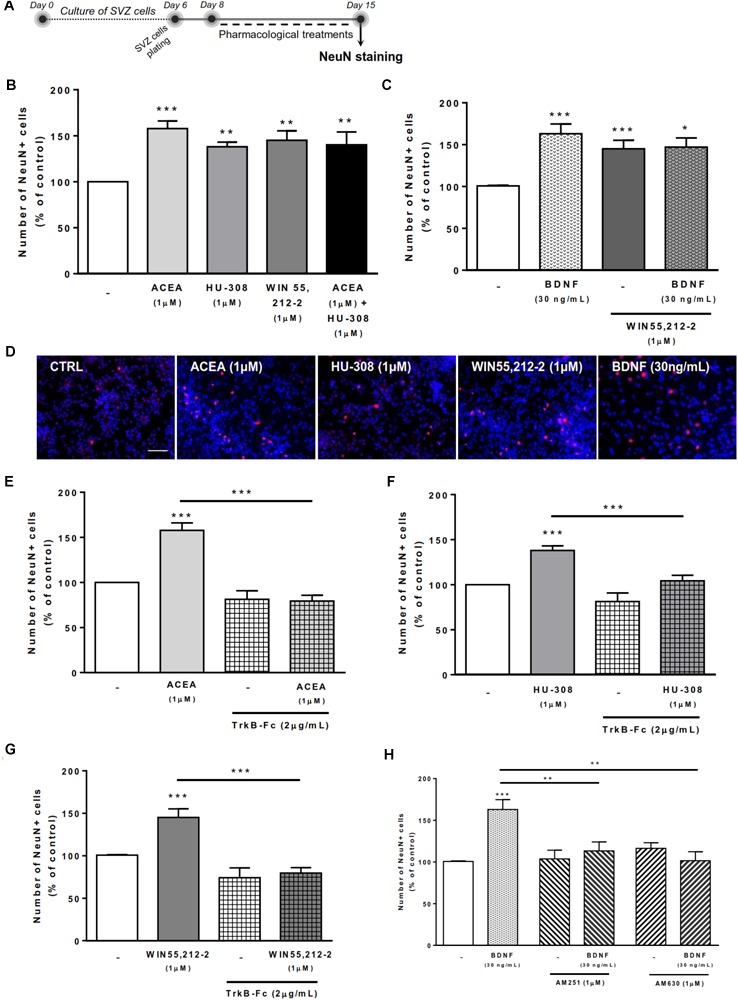
Cannabinoid receptor crosstalk with BDNF modulates SVZ neuronal differentiation. Cannabinoid receptor activation promoted SVZ neuronal differentiation, an effect dependent on the presence of endogenous BDNF. Similarly, BDNF effect upon SVZ neuronal differentiation was abolished by CB1R or CB2R antagonism. **(A)** Schematic representation of the experimental protocol. Day 0 represents the day of cultures; at Day 6 SVZ neurospheres were plated for 48h and at Day 8 cells were exposed to pharmacological treatments for further 7 days (Day 15). **(B,C,E–H)** Bar graphs depict the number of NeuN-positive cells. Values were normalized to the control mean for each experiment and are represented as mean ± SEM. Control was set to 100%. *n* = 5–24. ^∗^*p* < 0.05 and ^∗∗∗^*p* < 0.001 using Dunnett’s multiple comparison test. **(D)** Representative fluorescent digital images of cells immunopositive for NeuN (in red) and Hoechst 33342 staining (blue nuclei). Scale bar = 50 μm.

To evaluate the effects on SVZ neuronal differentiation, SVZ cells were treated with the test drugs in serum-free medium devoid of growth factors for 7 days (Figure 3A). As previously reported (Rodrigues et al., 2017), treatment of SVZ cells with selective agonists for CB1R and/or CB2R as well as treatment with non-selective cannabinoid receptor agonist, WIN 55,212-2, induced a significant increase in the number of NeuN-positive cells when compared to control cultures (Figures 3B–D). While testing the action of exogenous BDNF, we observed a significant increase in the percentage of NeuN-positive cells upon incubation with BDNF (163.0 ± 11.78% [95% CI: 138.4–187.6%]; *n* = 20, *p* < 0.001 vs. control) (Figures 3C,D). This effect persisted when cultures were co-incubated with BDNF together with non-selective cannabinoid agonist WIN 55,212-2 (147.1 ± 11.02% [95% CI: 121.7–172.5%]; *n* = 9, *p* < 0.05 vs. control) (Figures 3C).

Remarkably, in the presence of the BDNF scavenger (TrkB-Fc) none of the cannabinoid receptor agonists affected the percentage of NeuN-positive cells (*p* < 0.001 vs. agonists alone, Figures 3E–G). BDNF chimera scavenger (TrkB-Fc) alone was devoid of effect (*p* > 0.05 vs. control) (Figures 3E–G). These data indicate that endogenous BDNF is necessary for the actions of CB1R and CB2R upon SVZ neuronal differentiation.

Interestingly, the effect promoted by BDNF on SVZ neuronal differentiation was blocked when cells were co-incubated with either the CB1R selective antagonist AM251 (BDNF 30 ng/mL+AM251 1 μM: 113.3 ± 10.6% [95% CI: 89.2–137.4%]; *n* = 10, *p* < 0.01 vs. BDNF) (Figure 3H) or with the CB2R selective antagonist AM630 (BDNF 30 ng/mL+AM630 1 μM: 101.5 ± 10.8% [95% CI: 67.1–135.9%]; *n* = 4, *p* < 0.01 vs. BDNF) (Figure 3H). No significant alterations were found when incubating cultures with selective antagonists alone (Figure 3H).

Altogether the above results indicate that the effect of BDNF on SVZ neuronal differentiation is dependent on both CB1R and CB2R, while the effect of CB1R and CB2R is dependent on endogenous BDNF.

### BDNF-CB2R Interaction Regulates Self-Renewal in DG Cell Cultures

**FIGURE 4:**
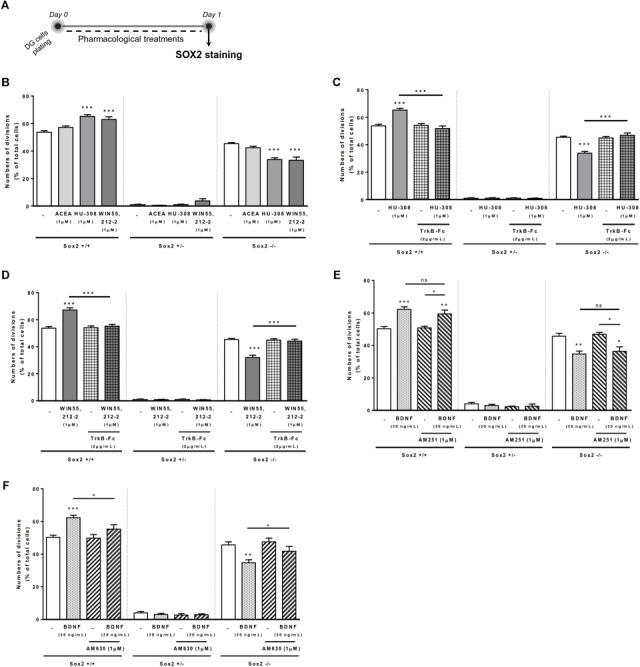
BDNF-CB2R crosstalk regulates DG self-renewal. CB2R selective and non-selective activation increased DG self-renewal capacity, an effect abrogated by endogenous BDNF removal. Conversely, BDNF-mediated increase in DG self-renewal was dependent on CB2R, but not CB1R, modulation. **(A)** Schematic representation of the experimental protocol used to study cell-fate. Day 0 represents the day of cultures where the DG cell suspension was treated with the drugs for 24 h. **(B–F)** Bar graphs depict the percentage of Sox2+/+, Sox2+/–, Sox2–/– cell pairs expressed as percentage of total cells per culture. Data are expressed as mean ± SEM. *n* = 3–7. *^∗^p* < 0.05, *^∗∗^p* < 0.01, and *^∗∗∗^p* < 0.001 using Dunnett’s multiple comparison test. ns, non-significant.

Since effects in SVZ may differ from effects on DG, both neurogenic niches having different functions (Bond et al., 2015), we repeated the above-mentioned experiments, but using DG cell cultures (Figure 4A). We firstly observed that although CB1R selective activation promoted no significant changes in the percentages of either Sox2+/+ or Sox2-/- cell pairs, CB2R selective activation with HU-308 or non-selective cannabinoid activation with WIN 55,212-2 induced a significant increase in the percentage of Sox2+/+ cell pairs (control: 53.70 ± 1.212% [95% CI: 50.5–56.8%]; HU-308 1 μM: 65.17 ± 1.35% [95% CI: 61.4–68.9%]; WIN 55,212-2 1 μM: 62.94 ± 2.02% [95% CI: 57.3–68.5%]; *n* = 3–5, *p* < 0.001) (Figure 4B), with a concomitant decrease in the percentage of Sox2-/- cell pairs (control: 45.35 ± 0.86% [95% CI: 42.9–47.7%]; HU-308 1 μM: 33.82 ± 1.23% [95% CI: 30.3–37.2%]; WIN 55,212-2 1 μM: 33.28 ± 2.31% [95% CI: 26.8–39.7%]; *n* = 3–5, *p* < 0.001 vs. control) (Figure 4B). This suggests modulation of DG cell-fate by CB2R selective activation, which was further tested by co-incubation with selective antagonists for CB1R and CB2R. Corroborating the involvement of CB2R, we observed that the effect mediated by CB2R selective agonist or by the non-selective CB1R/CB2R agonist in DG self-renewal was blocked by co-incubation with a CB2R selective antagonist, AM630 (1 μM), but not with a CB1R selective antagonist, AM251 (1 μM) (Supplementary Figure S1).

The presence of the BDNF scavenger, TrkB-Fc (2 μg/mL), abrogated both the increase in the percentage of Sox2+/+ cell pairs, and the concomitant decrease of Sox2-/- cell pairs, induced by either the CB2R selective agonist, HU-308 (1 μM; Figure 4C) or the CB1R/CB2R agonist, WIN 55,212-2 (1 μM; Figure 4D). This data clearly indicates that endogenous BDNF is important for CB2R-mediated control of DG cell-fate.

Exogenously added BDNF (30 ng/ml) increased the percentage of Sox2+/+ cell pairs (control: 50.31 ± 1.28% [95% CI: 47.17–53.45%]; BDNF 30 ng/mL: 62.25 ± 1.58% [95% CI: 58.38–66.12%]; *n* = 7, *p* < 0.001), and concomitantly decreased in the percentage of Sox2-/- cell pairs (control: 45.66 ± 1.85% [95% CI: 41.12–50.21%]; BDNF 30 ng/mL: 34.70 ± 1.79% [95% CI: 30.31–39.09%]; *n* = 7, *p* < 0.001) (Figures 4E,F). Remarkably, the action of BDNF on DG cell-fate was blocked by co-incubation with the CB2R selective antagonist AM630 (Figure 4F), but not by the CB1R selective antagonist AM251 (Figure 4E). No significant changes were found when incubating cultures with the selective receptor antagonists alone (Figures 4E,F). Overall, these data suggest that both BDNF and CB2R have a leading role in modulating DG cell-fate and that they reciprocally regulate each other actions.

### BDNF-CB2R Interaction Regulates Cell Proliferation in DG Cell Cultures

**FIGURE 5:**
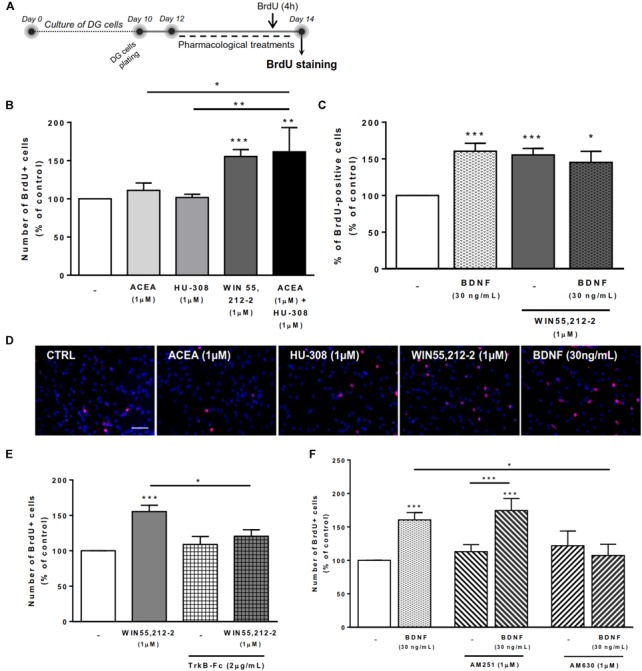
BDNF-CB2R interaction modulates DG cell proliferation. CB1R activation together with CB2R activation promoted DG cell proliferation, an effect dependent on the presence of BDNF. CB2R, but not CB1R, blockage abrogated BDNF-mediated increase in DG cell proliferation. **(A)** Schematic representation of the experimental protocol. Day 0 represents the day of cultures; at Day 10 DG neurospheres were plated for 48 h and at Day 12 cells were exposed to pharmacological treatments for further 48 h (Day 14). **(B,C,E,F)** Bar graphs depict the percentage of BrdU-positive cells. Values were normalized to the control mean for each experiment and are represented as mean ± SEM. Control was set to 100%. *n* = 6–17. ^∗^*p* < 0.05 and ^∗∗∗^*p* < 0.001 using Dunnett’s multiple comparison test. **(D)** Representative fluorescent digital images of cells immunopositive for BrdU (in red) and Hoechst 33342 staining (blue nuclei). Scale bar = 50 μm.

To assess the effects on DG cell proliferation, as we did before for SVZ cell proliferation, DG cells were treated with selective ligands for 48 h, incorporated BrdU was immunolabeled and positive nuclei percentage was determined (Figure 5A).

We could confirm (Rodrigues et al., 2017) that CB1R or CB2R selective activation with ACEA (1 μM) and HU-308 (1 μM), respectively, did not promote DG cell proliferation (Figures 5B,D). Interestingly, upon co-incubation with both selective cannabinoid receptor agonists (ACEA+HU-308) or with non-selective cannabinoid receptor agonist WIN 55,212-2 (1 μM), there was a significant increase in the number of BrdU-positive cells (ACEA 1 μM+HU-308 1 μM: 161.4 ± 31.85% [95% CI: 79.5–243.3%]; WIN 55,212-2 1 μM: 155.4 ± 8.89% [95% CI: 136.5–174.3%]; *n* = 13–17, *p* < 0.01 and *p* < 0.001 vs. control, respectively) (Figures 5B–D). These findings suggest that there is the need of a positive interaction between CB1R and CB2R for cannabinoids to affect cell proliferation at the DG.

Concerning the influence of exogenous BDNF, we observed a significant increase in the percentage of BrdU-positive cells upon incubation with BDNF (30 ng/mL; 160.4 ± 10.90% [95% CI: 137.0–183.8%]; *n* = 15, *p* < 0.001 vs. control) (Figures 5C,D) which persisted when co-incubation with the non-selective cannabinoid receptor agonist was performed (BDNF 30 ng/mL + WIN 55,212-2 1 μM: 145.2 ± 15.02% [95% CI: 103.5–186.9%]; *n* = 5, *p* < 0.05 vs. control) (Figure 5C).

Importantly, endogenous BDNF withdrawal from the media with TrkB-Fc blocked the WIN 55,212-2-mediated increase in BrdU-positive cells (WIN 55,212-2 1 μM + TrkB-Fc 2 μg/mL: 120.5 ± 9.21% [95% CI: 96.8–144.2%]; *n* = 6, *p* < 0.05 vs. WIN 55,212-2 alone) (Figure 5E) indicating that BDNF plays an important role on cannabinoid receptor-mediated DG cell proliferation. Interestingly, and similarly to what happens with DG cell-fate, the use of a CB2R selective antagonist was able to block the BDNF-mediated effect on DG cell proliferation (BDNF 30 ng/mL + AM630 1 μM: 107.3 ± 16.80% [95% CI: 53.8–160.8%]; *n* = 4, *p* < 0.05 vs. BDNF alone) (Figure 5F) while CB1R blockade did not affect the increase in BrdU-positive cells promoted by BDNF (BDNF 30 ng/mL+AM251 1 μM: 174.6 ± 17.84% [95% CI: 132.4–216.8%]; *n* = 8, *p* < 0.05 vs. BDNF alone) (Figure 5F). This suggests that CB2R plays an important role in modulating BDNF actions on DG cell proliferation, and that this action is independent of CB1R.

### BDNF Crosstalk With Cannabinoid Receptor Activation Modulates DG Neuronal Differentiation

**FIGURE 6:**
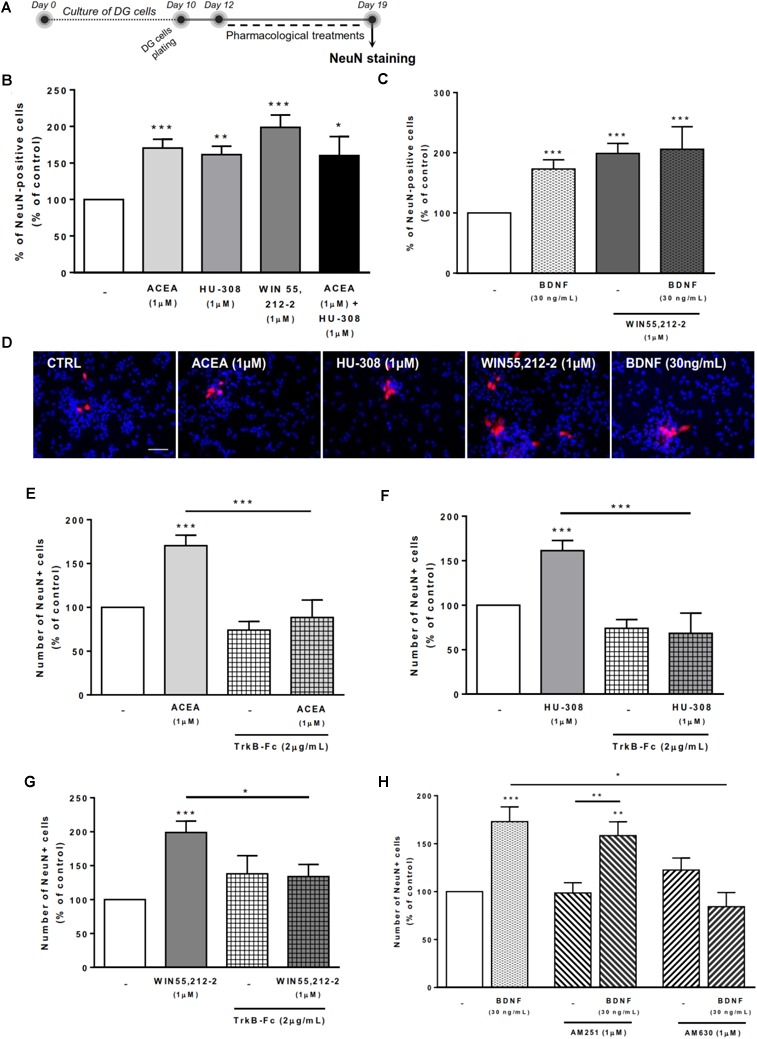
BDNF crosstalk with cannabinoid receptors tightly regulates DG neuronal differentiation. DG neuronal differentiation was increased upon cannabinoid receptor activation. BDNF was required for cannabinoid receptor-mediated effect on DG neuronal differentiation. CB2R, but not CB1R, antagonism blocked the effect promoted by BDNF upon SVZ neuronal differentiation. **(A)** Schematic representation of the experimental protocol. Day 0 represents the day of cultures; at Day 10 DG neurospheres were plated for 48 h and at Day 12 cells were exposed to pharmacological treatments for further 7 days (Day 19). **(B,C,E–H)** Bar graphs depict the percentage of NeuN-positive cells. Values were normalized to the control mean for each experiment and are represented as mean ± SEM. Control was set to 100%. *n* = 6–19. ^∗^*p* < 0.05 and ^∗∗∗^*p* < 0.001 using Dunnett’s multiple comparison test. **(D)** Representative fluorescent digital images of cells immunopositive for NeuN (in red) and Hoechst 33342 staining (blue nuclei). Scale bar = 50 μm.

Treatment of DG cells with all cannabinoid receptor agonists for CB1R and/or CB2R (Figure 6A) promoted a significant increase in the number of NeuN-positive cells when compared to control cultures (ACEA 1 μM: 170.4 ± 11.95% [95% CI: 143.4–197.5%]; HU-308 1 μM: 161.4 ± 11.42% [95% CI: 135.0–187.7%]; ACEA 1 μM+ HU-308 1 μM: 160.1 ± 26.07% [95% CI: 93.08–227.1%]; WIN 55,212-2 1 μM: 198.80 ± 16.74% [95 CI: 163.6–234%]; *n* = 9–19, *p* < 0.05, *p* < 0.01 or *p* < 0.001 vs. control) (Figures 6B–D), corroborating our previous data on the effects of cannabinoids on DG neuronal differentiation (Rodrigues et al., 2017).

We then investigated the role of exogenous BDNF administration in modulating DG neuronal differentiation. BDNF was shown to promote a significant increase in the number of NeuN-positive cells (173.0 ± 15.42% [95% CI: 140.1–205.9%]; *n* = 16, *p* < 0.001 vs. control) (Figures 6C,D), an effect that was maintained after co-treatment with non-selective cannabinoid receptor agonist (BDNF 30 ng/mL + WIN 55,212-2 1 μM: 205.9 ± 37.41% [95% CI: 114.4–297.4%]; *n* = 7, *p* < 0.001 vs. control).

Endogenous BDNF seems to be necessary for the actions cannabinoids upon DG neuronal differentiation, since the enhancement caused by CB1R or CB2R agonists in the percentage of NeuN-positive cells was prevented by co-incubation with the BDNF scavenger (Figures 6E–G).

Finally, we have observed that the effect promoted by BDNF on DG neuronal differentiation was blocked when cells were co-incubated with the CB2R selective antagonist AM630 (BDNF 30 ng/mL+AM630 1 μM: 84.3 ± 14.7% [95% CI: 20.8–147.8%]; *n* = 3, *p* < 0.05 vs. BDNF alone) (Figure 6H) but not with the CB1R selective antagonist AM251 (Figure 6H), indicating that CB2R is preponderant to modulate the action of BDNF on DG neuronal differentiation.

## Discussion

The present work reveals a yet not described interaction between BDNF and cannabinoid receptors (CB1R and CB2R) responsible to modulate several aspects of SVZ and DG postnatal neurogenesis. BDNF was shown to be an important modulator of SVZ and DG postnatal neurogenesis, its actions being under control of cannabinoid receptors. The relevance of each cannabinoid receptor to control the action of BDNF upon neurogenesis is different in the two neurogenic niches. While CB2R has a preponderant role in modulating BDNF actions on DG, BDNF-mediated SVZ postnatal neurogenesis is modulated by both CB1R and CB2R. A constant and clear finding in both neurogenic niches is that BDNF is required for cannabinoid actions to occur. It thus appears that a reciprocal cross-talk between cannabinoids and BDNF exist to modulate postnatal neurogenesis.

BDNF is a neurotrophin important in the regulation of several neuronal processes such as neuronal branching, dendrite formation and synaptic plasticity (Dijkhuizen and Ghosh, 2005; Gómez-Palacio-Schjetnan and Escobar, 2013). In line with this evidence, several studies have shed light on the actions of BDNF in the survival and differentiation of newborn neurons (Benraiss et al., 2001; Henry et al., 2007; Chan et al., 2008; Snapyan et al., 2009). Our findings now demonstrate that BDNF is able to affect early steps of postnatal neurogenesis, such as cell-fate, cell proliferation and neuronal differentiation of SVZ and DG cultures. We observed that BDNF promoted self-renewal of SVZ- and DG-derived cells as observed by an increase in self-renewal divisions, i.e., an increase in the percentage of Sox2+/+ cell-pairs. BDNF-CBR crosstalk has been reported to control several processes at the synaptic level (Zhao and Levine, 2014; Zhong et al., 2015) and we now extended these findings toward very early stages of postnatal neurogenesis. Interestingly, the increase in the SVZ and DG pool of stem/progenitor cells mediated by BDNF was fully abolished in the presence of CB2R antagonist but not CB1R antagonist. An exception is the influence of BDNF upon SVZ cell proliferation, which is not affected by CB1R or CB2R selective antagonism. In what concerns neuronal differentiation, both CB1R and CB2R are required for BDNF actions on SVZ whereas at the DG, only CB2R seem to affect BDNF-promoted neuronal differentiation. Overall, cannabinoid receptor blockade appears to influence more BDNF-induced actions upon early stages of DG neurogenesis in comparison to SVZ, highlighting the fact that cannabinoids distinctly modulate the effects promoted by BDNF in SVZ and DG neurogenesis.

It was previously known that the endocannabinoid system and cannabinoid receptors are important modulators of several stages of neurogenesis (Palazuelos et al., 2012; Xapelli et al., 2013; Prenderville et al., 2015; Rodrigues et al., 2017). In accordance with our previous data, SVZ and DG cells were differently affected by the same cannabinoid pharmacological treatments (Rodrigues et al., 2017). Considering cell fate, we observed that selective activation of CB2R activation promotes self-renewal of DG cells, but not of SVZ cells. This is consistent with several pieces of evidence showing a regulation of cell fate promoted by the activation of several signaling pathways [such as mitogen-activated protein kinase (MAPK) family (ERK, JNK and p38) and the phosphoinositide-3 kinase (PI3K)/AKT pathways] triggered by CBR activation (Molina-Holgado et al., 2007; Gomez et al., 2010; Soltys et al., 2010; Compagnucci et al., 2013).

On the other hand, our results reveal, for the first time, a role of cannabinoid receptors (CB1R and CB2R) in regulating DG cell commitment.

Considering cell proliferation, it is promoted by CB1R but not CB2 at SVZ, while at DG cell proliferation was only induced by co-activation of CB1R and CB2R. These results are in accordance with previous reports that have shown an increase in SVZ cell proliferation promoted by CB1R selective activation (Trazzi et al., 2010; Xapelli et al., 2013) and an increase in DG cell proliferation triggered by CB1R/CB2R non-selective activation (Aguado et al., 2005; Rodrigues et al., 2017). Importantly, while we also detected an effect with the non-selective CB1R/CB2R agonists, none of the selective agonists when applied in the absence of the other agonist were effective to promote cell proliferation in the DG, highlighting the need of caution while interpreting negative results with each of those agonists separately.

Regarding neuronal differentiation, our data indicate that in SVZ and DG neurogenic niches both subtypes of cannabinoid receptors are able to promote neuronal differentiation. These data are in accordance with previous reports in which cannabinoid receptor activation enhanced neuronal differentiation of NSPC by CB1R- (Compagnucci et al., 2013) or CB2R-dependent (Avraham et al., 2014) mechanisms.

The most important finding in the present work is that most of the cannabinoid-induced effects upon cell proliferation and neuronal differentiation depend on the presence of BDNF, suggesting the existence of a BDNF-endocannabinoid feedback loop responsible for regulating these processes. Previous reports have shed light on the existence of a putative interaction between BDNF and cannabinoid receptors (Howlett et al., 2010), but none focused upon neurogenesis. De Chiara et al. (2010) have identified a novel mechanism by which BDNF mediates the regulation of striatal CB1R function. Moreover, others have suggested that BDNF can regulate neuronal sensitivity to endocannabinoids through a positive feedback loop important for the regulation of neuronal survival (Maison et al., 2009). Evidence also shows the involvement of BDNF in the actions mediated by cannabinoids against excitotoxicity (Khaspekov et al., 2004), in synaptic transmission and plasticity (Klug and van den Buuse, 2013; Zhao et al., 2015; Yeh et al., 2017) and in several behavioral outputs (Aso et al., 2008; Bennett et al., 2017). Previous animal studies have shown that acute (Derkinderen et al., 2003) and chronic (Butovsky et al., 2005) Δ^9^-THC (major psychoactive constituent of cannabis; CB1R and CB2R agonist) administration is associated with an increase in BDNF gene expression. Moreover, it was shown that overexpression of BDNF is able to rescue cognitive deficits promoted by Δ^9^-THC administration in a mouse model of schizophrenia (Segal-Gavish et al., 2017). In human studies it was found that Δ^9^-THC increased serum BDNF levels in healthy controls, but not in chronic cannabis users (D’Souza et al., 2009). In fact, cyclic AMP response element-binding protein (CREB) may be the common linking element because it is an important regulator of BDNF-induced gene expression (Finkbeiner et al., 1997), and has been reported to control several steps of the neurogenic process in the adult hippocampus (Nakagawa et al., 2002) and SVZ (Giachino et al., 2005). Consistently, cannabinoids have been shown to induce CREB phosphorylation (Isokawa, 2009) and also to promote changes in BDNF and CREB gene expression (Grigorenko et al., 2002). In addition, the work done by Berghuis et al. (2005) showed that endocannabinoids stimulate TrkB receptor phosphorylation during interneuron morphogenesis. Most importantly, in the same study, the authors observed by co-immunoprecipitation the formation of heteromeric complexes in PC12 cells expressing TrkB receptors and CB1R (Berghuis et al., 2005). Our study brings new and relevant information on the interaction between cannabinoid receptors and BDNF in controlling SVZ and DG neurogenesis, and clearly highlights that this interaction is reciprocal. In fact, neurogenesis promoted by cannabinoid receptor activation depends on the presence of endogenous BDNF, while the effects mediated by BDNF upon neurogenesis are directly regulated by modulation of CB1R or CB2R.

Although our study is based on an *in vitro* approach, the neurosphere assay, it represents a highly relevant model. *In vitro* systems of NSPC allow an easier access and better control of experimental variables as well as a thorough analysis of mechanisms happening at cellular and molecular level providing useful information to be further validated *in vivo* (Singec et al., 2006). Moreover, the heterogeneous composition of the NSPC grown in neurospheres is extremely relevant because it holds some of the features, such as close contact with neighboring cells (newly generated neuroblasts, astrocytes and oligodendrocytes), that resemble those of the physiological niche (Casarosa et al., 2014). These well-established advantages (Aguado et al., 2007; Agasse et al., 2008; Azari et al., 2010) are the reason why we have used this *in vitro* approach to study the intrinsic properties of NSPC and to understand the interaction between BDNF and cannabinoids in modulating neurogenesis. It is, however, important to mention that the mechanisms governing the regulation of neurosphere dynamics might be different from the ones regulating *in vivo* adult neurogenesis (Casarosa et al., 2014). Indeed, further *in vivo* studies will be required to comprehensively understand the role of BDNF in regulating the actions of cannabinoid receptors on postnatal neurogenesis.

Taken together, our data highlight a novel level of complexity for the regulatory mechanisms involved in NSPC dynamics, which involve the interplay of multiple signaling cues, and where BDNF and cannabinoids may play a relevant role. Further *in vitro* studies are required to detail the molecular mechanisms involved, as well as *in vivo* studies to determine the functional consequences of the BDNF/cannabinoid crosstalk to control neurogenesis. Nevertheless, our study provides evidence for the need of integrative strategies whenever focusing on NSPC for brain repair.

## Author Contributions

FF, FR, and RR: conception and design of the work, acquisition, analysis and interpretation of data for the work; drafting and revising critically the work for important intellectual content and final approval of the version to be published. AS: conception and design of the work; interpretation of data for the work; revising critically the work for important intellectual content and final approval of the version to be published. SX: conception and design of the work, acquisition, analysis and interpretation of data for the work; drafting and revising critically the work for important intellectual content and final approval of the version to be published.

## Conflict of Interest Statement

The authors declare that the research was conducted in the absence of any commercial or financial relationships that could be construed as a potential conflict of interest.

## Supplementary Material

FIGURE S1 CB2R modulation regulates DG cell-fate. Non-selective cannabinoid receptor activation or selective CB2R activation increased DG self-renewing capacity and these effects were dependent on CB2R. **(A–D)** Bar graphs depict the percentage of Sox2+/+, Sox2+/-, Sox2-/- cell pairs expressed as percentage of total cells per culture. Data are expressed as mean ± SEM. *n* = 5–7. ^∗^*p* < 0.05, ^∗∗^*p* < 0.01 and ^∗∗∗^*p* < 0.001 using Dunnett’s multiple comparison test.

## Abbreviations

Δ^9^-THC: Δ^9^-tetrahydrocannabinol
BDNF: brain-derived neurotrophic factor
CB1R: cannabinoid receptor type 1
CB2R: cannabinoid receptor type 2
CNS: central nervous system
DG: dentate gyrus
EGF: epidermal growth factor
FGF-2: fibroblast growth factor-2
NSPC: neural stem/progenitor cells
SGZ: subgranular zone
SVZ: subventricular zone
