# Supplementary figures and images for: Brain-Derived Neurotrophic Factor (BDNF) Role in Cannabinoid-Mediated Neurogenesis
Source: Front Cell Neurosci. 2018 Nov 28;12:441. doi: 10.3389/fncel.2018.00441 (PMC6279918; doi:10.3389/fncel.2018.00441)

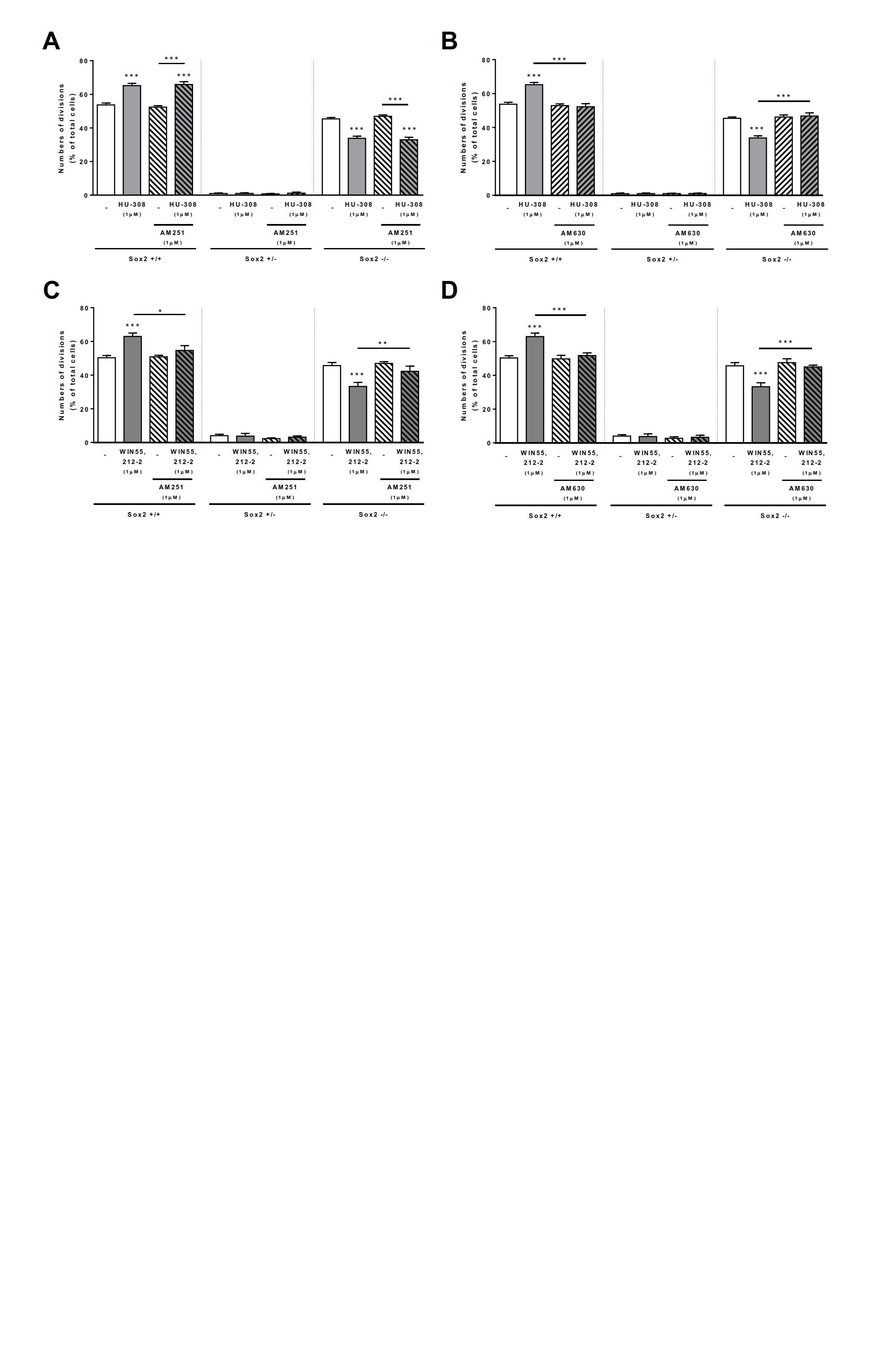

Supplement: FIGURE S1 — CB2R modulation regulates DG cell-fate. Non-selective cannabinoid receptor activation or selective CB2R activation increased DG self-renewing capacity and these effects were dependent on CB2R. (A–D) Bar graphs depict the percentage of Sox2+/+, Sox2+/-, Sox2-/- cell pairs expressed as percentage of total cells per culture. Data are expressed as mean ± SEM. n = 5–7. ∗p < 0.05, ∗∗p < 0.01 and ∗∗∗p < 0.001 using Dunnett’s multiple comparison test. [file Image_1.TIF]
